# A Radar-Enabled Collaborative Sensor Network Integrating COTS Technology for Surveillance and Tracking

**DOI:** 10.3390/s120201336

**Published:** 2012-01-31

**Authors:** Robert Kozma, Lan Wang, Khan Iftekharuddin, Ernest McCracken, Muhammad Khan, Khandakar Islam, Sushil R. Bhurtel, R. Murat Demirer

**Affiliations:** 1 Center for Large-Scale Integrated Optimization & Networks (CLION), FedEx Institute of Technology, University of Memphis, 365 Innovation Drive, Memphis, TN 38152, USA; E-Mails: lanwang@memphis.edu (L.W.); emccrckn@memphis.edu (E.M.); mmkhan@memphis.edu (M.K.); kmislam@memphis.edu (K.I.); srbhrtel@memphis.edu (S.R.B.); 2 Department of Electrical and Computer Engineering, Old Dominion University, Norfolk, VA 23529, USA; E-Mail: kiftekha@odu.edu; 3 Computer Engineering Department, Istanbul Kultur University, E5 Atakoy Campus E5 Otoyolu Londra AsfaltÄ±, BakÄ±rkoy, 34156 Istanbul, Turkey; E-Mail: rmuratdemirer@gmail.com

**Keywords:** Doppler radar, wireless sensor mote, autonomous sensor network, surveillance, tracking

## Abstract

The feasibility of using Commercial Off-The-Shelf (COTS) sensor nodes is studied in a distributed network, aiming at dynamic surveillance and tracking of ground targets. Data acquisition by low-cost (<$50 US) miniature low-power radar through a wireless mote is described. We demonstrate the detection, ranging and velocity estimation, classification and tracking capabilities of the mini-radar, and compare results to simulations and manual measurements. Furthermore, we supplement the radar output with other sensor modalities, such as acoustic and vibration sensors. This method provides innovative solutions for detecting, identifying, and tracking vehicles and dismounts over a wide area in noisy conditions. This study presents a step towards distributed intelligent decision support and demonstrates effectiveness of small cheap sensors, which can complement advanced technologies in certain real-life scenarios.

## 1. Introduction

Historically, wireless surveillance systems have used infrared, acoustic, seismic and magnetic signals for passive sensing, and optics and ultrasonic signals for active sensing. Radar has been conspicuously absent from wireless surveillance systems [1], particularly because radar systems are conventionally bulky and have high energy consumption. With the advent of the micro-power pulse radar in the 90’s [2], low power radar becomes a possibility. Subsequently, technical progress in sensor networks has prompted efforts to integrate radar as one of the sensor modalities to autonomous sensor network platforms [1,3]. An autonomous sensor network is a collection of sensor nodes with limited processing, power, and communication capabilities that monitor a real world environment through differing modalities. The nodes gather information about the local environment, preprocess the data, and transmit the output via wireless channels to a base station. The base station may broadcast commands to all or some of the sensor nodes in the network. Using intelligent framework design, the network can support decision-making and provide the capability to detect, track, and identify targets over a wide area.

Recent research developments include designing systems using Mica2 mote and 2.4 GHz TWR-ISM-002 radar sensor from Advantaca [4]. The integration includes a custom designed power board that provides power required for the radar, onboard antenna, and an optional Mica sensor board. BumbleBee is another integrated radar mote sensor developed by the Samraksh Company [3], which includes a low-power Pulsed Doppler Radar (PDR) and a TelosB mote [5]. The key features of BumbleBee include: detection range between 1 m to 10 m that is controllable via software, on-board internal antenna, 60 degree conical coverage pattern, and detection of radial velocities up to 2.6 m/s. The radars used in the above-mentioned platform offer the detection of movement and the velocity of a moving target. BumbleBee has the capability to compute range with additional post-processing processing methods; however, it is not measured simultaneously with velocity.

It is desirable to have low-cost radar-mote that measures the range and velocity simultaneously with simple signal processing techniques, and having more functionality, such as target classification and tracking. To achieve this goal, we introduce a sensor system consisting of a variety of sensor motes with radar, light, acoustic, and vibration sensors, which can be used for autonomous wireless surveillance and sensing. Compared with previous work on wireless radar motes [1,3], our multi-modal sensor system not only detects targets, but also produces accurate range and velocity estimates of the targets simultaneously with simple signal processing techniques. Moreover, our system can use various complementary sensors to classify targets based on such characteristics as shape, material, and weight [6]. In addition, our radar can produce continuous estimates of range and velocity, thus enabling us to track target movements.

Each sensor modality has its advantages and disadvantages. Acoustic sensors can be used to distinguish objects with different sound signatures, e.g., acoustic sensor systems have been used to classify vehicles. To a certain extent, acoustic sensors can also provide information related to direction of motion, speed and location. However, they are highly susceptible to background noise and wind conditions. Vibration sensors can distinguish objects with different weights and speeds, but these sensors are also susceptible to environmental disturbances. Radars can distinguish different materials and shapes, while providing estimations of speed and location. However, radar cannot differentiate between similar objects with different weights or acoustic signatures. By combining the data from acoustic, vibration and radar sensors, we can increase the accuracy of speed and location estimation. Furthermore, combining the sensor modalities enlarges the set of features used for object classification, thus widening the range of objects that can be classified by the system and making our results more reliable, as demonstrated by our experiments.

In this work, we describe the integration of low-cost (<$50 US) miniature low-power radars with wireless motes. We then describe the detection, ranging and velocity estimation capabilities, classification, as well as the tracking features of the mini-radar mote. We also demonstrate the integration of the radar and other sensing modalities, and discuss the possibilities of developing a robust pervasive sensor system using multi-modal technology.

## 2. Description of the Multi-Sensor Monitoring System

### 2.1. Miniature Low-Power Radar

**Figure 1.**
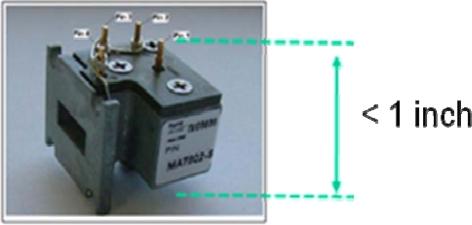
Miniature M/A-COM MACS-007802-0M1RSV K-Band Doppler transceiver [7].

This section describes the small, low-cost K-band radars that we use to detect and characterize targets. The RF transceivers are MACS-007802-0M1RSV manufactured by M/A-COM (Tyco Electronics, Figure 1) [7], and are used primarily for automotive applications, e.g., front and rear-end collision detection, ground speed measurements, as well as motion detection, e.g., automatic door openers. We have also used MACS-007802-0M1R1V from the same manufacturer, which has a slightly higher output power than MACS-007802-0M1RSV. The radar utilizes a Gunn diode oscillator and transmits a continuous wave at 24.125 GHz. It also has 0.3 GHz of bandwidth that can be controlled by applying external voltage, making it capable of estimating target range and velocity. The low-power consumption and small size make these devices excellent candidates for autonomous, compact sensors in a distributed network.

An external voltage pulse ramp using a waveform generator is applied to the radar, which emits a continuous frequency modulated K-band signal with 300 MHz bandwidth. The received energy is internally mixed with the transmitted signal and low-pass filtered to supply in-phase and quadrature output components on separate pins. Fast Fourier Transform (FFT) is performed over samples within the chirp (fast-time) to extract range information about a target. A second FFT is performed over a series of chirps (slow-time) to estimate the velocity of the target. Table 1 shows relevant radar parameters [7].

**Figure 2.**
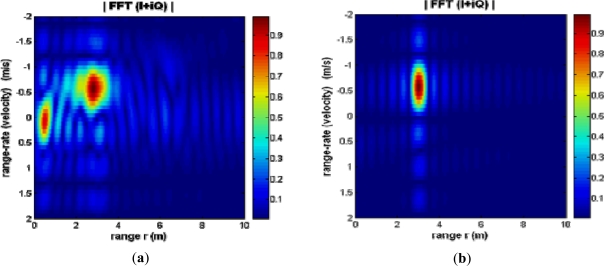
Range-rate (velocity, m/s) *vs*. range (m) intensity plot of a dismount approximately at 3 m range, as it walks towards the radar transceiver. (**a**) Result with actual dismount movement measured by the radar; (**b**) Simulated version of this scenario, with point targets in the calculations.

Figure 2 demonstrates results of a simple moving dismount experiment performed with the radar. A small K-band antenna was secured to the waveguide flange of the radar. The radar was supplied with a ramp waveform (between 0.5 and 10 volts) at a frequency of 1 KHz, and a human dismount walked toward the radar at velocity of approximately 0.5 m/s. At a target range of 3 m, the complex output waveforms of the radar were captured on an oscilloscope over 10 ramp cycles (chirps). The recorded data were processed and consequently displayed in the form of the range-rate (velocity) *vs.* range intensity plot, see Figure 2(a). As seen in Figure 2, the energy is concentrated at the location representing scattering from the moving dismount, *i.e.*, the max is at values of range 3 m and velocity 0.5 m/s. The range-rate (velocity) has maximum in the negative region of the graph as the target is moving toward the radar and distance was decreasing. The reverse situation is observed when the target moves away from the radar. Experiments have been completed to systematically evaluate the performance of the radar, in combination with other sensor nodes, as described in consecutive sections.

### 2.2. SBT80 Sensor Board and TelosB Mote

In our multi-modal sensor system, radar data are supplemented with data obtained using SBT80 sensor motes [8] developed by EasySen. The SBT80 sensor board contains several sensor modalities that can be used for detection and classification purposes. It includes a visual light sensor that we use to trigger other sensors. For our experiments, the visual light sensor is preferable since it detects objects tripping its sensor fairly precisely. In real world situations, these sensors may be replaced by passive infrared sensors. An acoustic sensor provides a raw audio waveform of the surrounding area. A dual axis accelerometer provides vibration information that can be used to infer mechanical characteristics of the target. The SBT80 also includes a dual-axis magnetometer and a temperature sensor, which were not used in the present experiments. An overview of the sensor modalities is given in Table 2. Each SBT80 sensor board sits atop a TelosB mote [5] capable of storing, processing and transmitting sensor data.

The TelosB mote is developed by UC Berkeley for low-power research development. Its main components are an 8 MHz 16-bit microcontroller, an IEEE 802.15.4 radio with integrated antenna, a 1 MB serial flash for data logging, and a 12-bit ADC with 8 channels. It supports the TinyOS operating system [9] and it is programmable through an onboard USB interface using the nesC language [10]. The mote has very low power consumption: the microcontroller draws 1.8 mA of current when active and 5.1 μA when asleep, and the RF transceiver draws 23 mA, 21 μA, and 1 μA of current in receive, idle and sleep mode, respectively. Powered by 2 AA batteries, the mote is expected to last weeks with moderate usage. Such energy efficiency, however, comes with severe resource constraints. The microcontroller, MSP430 from Texas Instruments, has only 48 K bytes of program flash memory and 10 K bytes of RAM. The limited memory size presents a significant challenge to our design as the radar and other sensors produce a large amount of data periodically. In order to provide high-quality sensor data without overflowing the RAM, we move the sensor data from the RAM to the larger serial flash after a specified number of runs (20 in our experiments). When the serial flash in a mote is reaching its capacity limit, we transfer the data from the mote to our base station through the mote’s wireless radio, which uses the 2.4 to 2.4835 GHz ISM band and has a transmission rate of 250 kbps. The onboard antenna has an outdoor transmission range of 75 to 100 meters and an indoor range of 20 to 30 meters. If the base station is outside the transmission range of the motes, we can run a multi-hop routing protocol on the motes to deliver the data. The mote has a 6-pin and a 10-pin connector to interface with additional devices, such as analog sensors and LCD displays. The SBT80 sensor unit uses these connectors to attach to the TeloB mote.

### 2.3. Integration of Radar and Wireless Mote

The SBT80 sensor boards require no modification to work with the TelosB mote [5] as they directly plug into the TelosB platform. However, the miniature Doppler radar was not built to such specifications. In order to retrieve data from the radar, we designed an additional electrical circuitry to integrate the radar and the TelosB mote platform. As a result, we successfully integrated the two independently manufactured devices.

**Figure 3.**
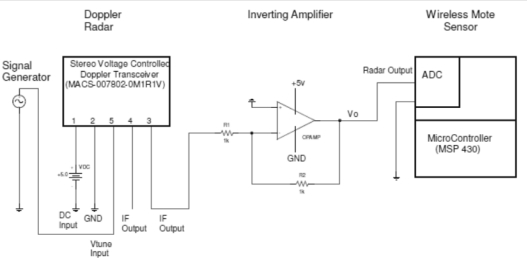
Diagram of radar integrated with the wireless mote.

Figure 3 shows the hardware setup of our integrated radar-mote system. A power supply provided the required 5 volts to the radar and to the amplifier between the radar and mote. The mote is powered by two AA batteries. The signal generator supplies a 1 kHz ramp wave ranging from 5 V to 10 V to tune the output frequencies [f_0_,f_1_] = [24.0,24.3] GHz of the Doppler radar transceiver.

When we physically connected our components together, we found that the radar outputs a negative voltage, but the mote only accepts positive voltage input. Thus we introduced one inverting one non-inverting amplifier circuits between the output of the Doppler radar and the mote’s input port, one for the I channel and the other for the Q channel. The inverting amplifier circuits invert the input signal with a gain of 5 and they were built using off-the-shelf components. This solution worked well in the present experiments.

The integrated system must provide reliable and accurate reading of the actual signal from the radar using the mote. The mote ADC has a 12-bit resolution. A series of reference experiments using a standard lab oscilloscope has confirmed that the accuracy was indeed sufficient for our goals. We managed to achieve the sampling frequency of 200 kHz by tuning the programmable parameters of the mote ADC using standard TinyOS data structures and interfaces. For our experimentation we sample the Doppler radar at 200 kHz and take 2,000 samples, illuminated with a train of 10 chirp pulses. We sample the vibration and acoustic sensors at 8 kHz and also take 2,000 samples from each sensor. In the above setup, the radar receives its tuning voltage input from a stand-alone signal generator and power from a separate power source. However, on-board chips can serve these purposes and we are testing such a design in our on-going experiments.

### 2.4. Signal Processing Architecture and Software

**Figure 4.**
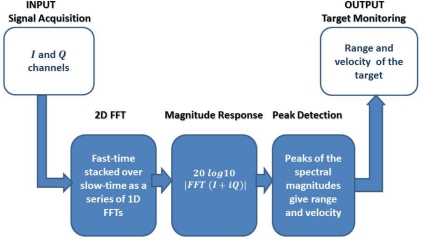
The general architecture of the radar signal processing using I and Q input channels. The output includes the velocity and the distance of the target.

Our sensor software is a three-layered set of modules written in various languages to facilitate data collection and analysis. In the case of radar motes, channels I and Q channel data are recorded in order to obtain the target range and velocity information, see Figure 4. Our goal is to make a smooth transition from raw mote data collection to a Matlab-based signal processing tool. The layers we used are the sensor mote layer, base station layer, and the analysis layer. Each layer abstracts a portion of the implementation for modularity. Communication between layers is done through network interfaces. The layers are described as follows:

- The first layer is the *mote layer*, which performs the actual sensing. The software at this layer is written in the nesC language [10] and executed in TinyOS environment [9]. TinyOS is an operating system environment designed for small embedded systems in which resources are highly constrained. nesC is a component-based language and uses syntax similar to C. Motes are programmed via a USB connection to a PC. We developed a sensing software program for two types of motes. The sentry sensor-mote monitors its visual light sensor and detects when shadows of objects pass by it. When motion is detected it broadcasts an ‘Alert’ message via its radio transmitter. ‘Alert’ messages are sent to other sensor-motes in the area that then began to sense their environment. Sensor-motes run a separate sensing program designed to sample sensors at high speeds for a short amount of time. Their clocks are synchronized with the sentry mote. Here our main constraint is the size of memory available. The TelosB mote hardware used by the sensor-motes has only 10 KB of RAM. Each capture run is saved onto the flash memory and, when all runs are finished, this data is transmitted to the second layer running on a base station composed of a PC and a mote attached to the PC.
- The *base station layer* includes several applications written for the base station. First, data is immediately received by the base station application running on the mote attached to the PC. Second, a Serial Forwarder application on the PC receives data from the base station application via USB and sends this to our service component written in Java. The service layer tests the validity of the data by checking for lost packets and requests retransmission for any incomplete data sets. This layer also has some control functionality. Users can change sampling periods and the number of runs per experiment from the Java console.
- Data from the mote are available to the *analysis layer* written in Matlab. Matlab can instantiate Java objects that we use to contact the service layer. Thus in the Matlab environment a user only needs to create a Java object and read raw mote data from that object. At this layer the user can perform any required signal processing techniques.

## 3. Experimental Setup and Results

### 3.1. Experimental Procedure

**Figure 5.**
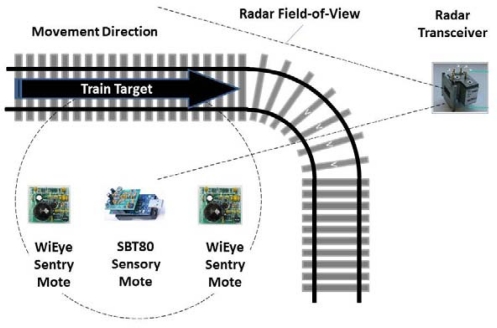
Experimental arrangement for the autonomous distributed sensor network, including triggering, acoustic, vibration, and radar I channel motes; mote Q is not shown.

This section describes the experimental procedure and results obtained for target range/velocity estimation and object tracking. Experiments have been conducted with various target configurations using two model locomotive trains as moving targets, called Train A and Train B, respectively. We also experimented with Trains having different payloads in order to study the sensitivity of our approach to various object configurations. In each experiment run, one of the two trains travels in a loop around an oval track, in which linear segments have been included for calibration purposes, see Figure 5. At the end of the straight segment at one side of the track, we marked distances at regular intervals of 0.8, 1.2, 1.6 and 2 m from the radar, and sensory data are captured at these marks.

A sentry mote is placed at each mark along with a light source. As the object passes between the sentry and the light source, it will decrease the amount of light reaching the sentry’s visual light sensor. This process is used for triggering the motes with SBT80 sensor boards and radar-motes. The SBT80 motes are used to collect data for acoustic and vibration modalities. Two radar-motes are used to collect data from channel I and channel Q of the radar, respectively.

Train A has adjustable speed controllable by a unit between level 100 and 40, which correspond to the maximum and minimum speed implemented in the present experiments. These levels corresponds to maximum Train velocity of 0.798 ± 0.078 m/s. (dial 100) and minimum velocity of 0.469 ± 0 m/s (dial 40), respectively.

Using the specified distances and the measured time to pass between the marked positions, the average actual velocity of the train has been determined during the given time period. Comparing actual velocities and results of Doppler velocity measurements, the accuracy of radar measurements has been evaluated. Various reflectors are mounted on the trains with three types of materials (paper, plastic, and metal), and two different shapes (tetrahedron and flat rectangular), in order to test the classification performance of the system.

Experimental runs are triggered by the target passing by the photo sensor. In every run 2,000 data points have been captured by the motes. We employed a sampling frequency of 8 kHz for vibration and acoustic nodes, and 200 kHz for the radar. Each mote saves its 2,000 sample point buffer to flash memory for a specified number of runs (typically 20). Once all runs are completed, the motes transmit their payloads to a base station, which saves those data to a disk. The base station can also send messages to other motes, which enables us to configure parameters, such as sampling frequency and number of runs to take during runtime. The obtained signals are further processed for range-velocity estimation and classification at the base station.

We gather four data sets from our experiments. The first set includes all combinations of speed, distance, and reflection profiles for one model train. A second smaller set is gathered from the same train but with multiple rail carts. A third set includes additional data taken from a different model train with only a single speed setting. Finally, in the fourth set, when the motes are triggered they capture several snapshots of the target consecutively rather than waiting for a second trigger. We use this data set to track the target as it travels past the radar transceiver. Table 3 summarizes our experimental parameters.

### 3.2. Range and Velocity Estimation Using Radar-Mote

**Figure 6.**
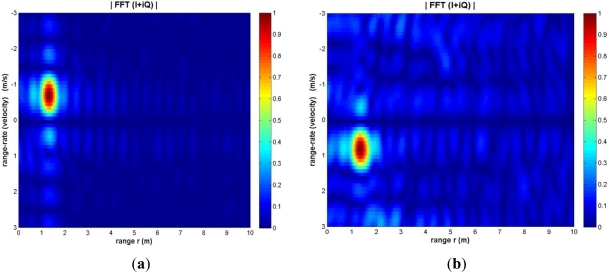
Examples of range-velocity intensity plots as the target was moving towards and away from the radar at a distance of 1.2 m at a speed of 0.69 m/s. The front of the target was covered with a metallic tetrahedron reflector. (**a**) Case of forward movement; (**b**) Backward movement.

We have conducted experiments with a variety of conditions as shown in Table 2 and determined the target range and velocity using data of the Doppler radar-mote. Figure 6 shows examples of range-velocity intensity plots when the target was moving towards and away from the radar at a distance of 1.2 m with a speed of 0.69 m/s (dial position 80 on the control unit). Negative velocity indicates that the target is moving towards the radar.

Table 4 summarizes the estimated range and velocity of the target using Doppler radar measurements for all experimental configurations. Each entry in Table 3 gives the average range and velocity of 20 runs with the standard deviation of the runs. The table also shows the range as knows from the markings of the triggering mote position, as well as the velocity independently determined from known distance and time stamp measurements. We conclude that radar measurements determine the range and velocity with good accuracy in all experiments. There is an increased error in the case of the lowest velocity value of 0.4 m/s, which is attributed to the actual variation and of the train movement during slower lower velocities.

### 3.3. Target Tracking

We tested the integrated wireless Doppler radar system to track a target moving within the field of view of the radar transceiver. We checked whether the radar can track the changes in range and velocity dynamically. In a specific experiment, we started to track the target starting at a distance of 2.3 m from the receiver. The velocity of the train was 0.74 m/s, *i.e.*, the max velocity corresponding to dial position D = 100. We captured four snapshots while the train was moving at the given velocity toward the radar mote.

**Figure 7.**
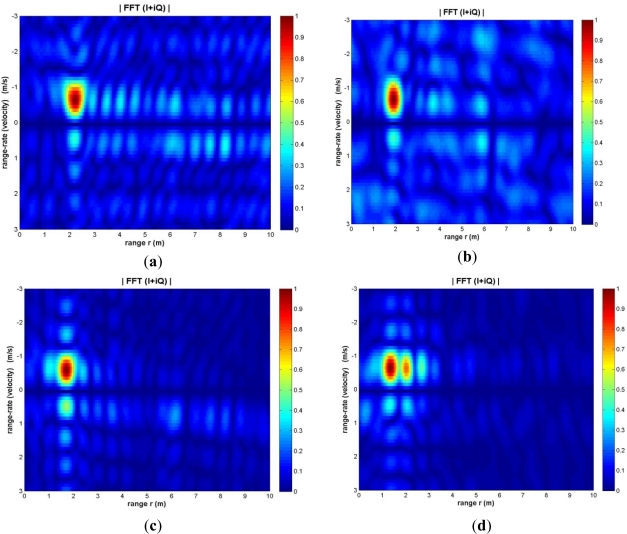
Range and velocity intensity plots for target tracking experiment: (**a**) Capture 1; (**b**) Capture 2; (**c**) Capture 3; (**d**) Capture 4.

The results are illustrated on the range-velocity intensity plots in Figure 7. Based on the Doppler signatures, the range and velocity has been estimated, see Table 5. Except for the first capture, the train is moving toward the radar at a velocity close to the expected constant value (0.75 m/s). The range is in general decreases at a constant rate, except for the first capture. The velocity in capture 1 is lower than the other snapshots. This discrepancy is attributed to the fact that during the first capture the train was moving at the curved section of the track, and had lower speed. These preliminary results of tracking are encouraging and further elaborate tracking experiments are underway.

## 4. Integration of Multi-Sensory Information for Target Identification

### 4.1. Classification Algorithms

Multiple Signal Classification (MUSIC) algorithm is used for spectral density estimation of all sensory modalities to allow for a unified data processing and data integration.

MUSIC estimates the eigenvectors of the sample correlation matrix and it uses the pseudo-spectrum estimate of the mean corrected input signal for each sensor at normalized frequencies at which the pseudo spectrum is evaluated [11–13]. After applying the MUSIC algorithm for signal processing, we classify the estimated power spectra.

To demonstrate the classification capability of our system, we use Weka [14] as a classification tool on the collected data. We run our data through three classifiers: Support Vector Machines, Multi-Layer Perceptron, and Random Forest:

- *Support Vector Machines (SVMs)* are a set of supervised learning methods used for classification and regression analysis.
- *Multi-Layer Perceptrons (MLPs)* are feed-forward neural networks that are trained with the standard back propagation algorithm. MLPs are widely used for pattern classification.
- *Random forest* is an ensemble classifier. It consists of many decision trees and outputs the classification that has the most votes over all the trees. It maintains good accuracy when portion of data is missing. However, it is prone to over fitting in some cases.

### 4.2. Results of Classification Using Vibration, Acoustic and Radar Data

In this section, results of classification are presented using vibration, acoustic and radar data. We process power spectral densities of sensor data obtained by MUSIC algorithm using normalization and Principal Component (PC) filters in Weka software package [14]. We compared three classifiers: Random Forest, MLP and SVM. Weka produces a number of metrics for evaluating the performance of the classifiers used. The “Correctly Classified” metric is self-explanatory. The ROC curve is a plot of the true positive rate against the false positive rate for the different possible cut points of a diagnostic test. It shows the tradeoff between sensitivity and specificity. The area under the curve (ROC area) is a measure of test accuracy. Kappa statistics indicate whether predictions and actual classes are correlated.

In Table 6, the performance of the acceleration (vibration) sensor is presented. From Table 6 it is seen that for all three experiments the classifiers were able to distinguish with good accuracy between different trains, different speeds, as well as between single train or a train with multiple railcars. This conclusion is true with the exception of SVM for the multiple cars experiment, where only 60% classification accuracy is obtained with SVM. The observed degradation of the performance is most likely due to the small data set being used (40 experiments). The ROC Area shows that in general there is high test accuracy for vibration data. Kappa statistic shows that the predictions provided by vibration data are stable, with the exception of SVM for multiple cart experiments.

We present the results of acoustic classification in Table 7. Two observations can be made from this Table. First, we can successfully classify two different trains when they are moving, as the trains create different acoustic signals. Second, we can also classify different speeds of the moving trains. The ROC Area and Kappa Statistics also support these findings.

Vibration or acoustic data do not allow us to differentiate among different reflectors, as those signals are not influenced by electromagnetic properties of the target. For that classification, we can use radar data to complement other sensor modalities. We have obtained radar data using three different configurations, in which the target train moved with reflectors of different shape and material. For each of the three configurations, 640 runs were used for classification purposes.

The results with radar data are presented in Table 8. In the first configuration, we used metal and paper reflectors of the tetrahedron shape to test if the radar can differentiate between different types of material. For this case, MLP produced the best classification result among the three classifiers. By applying MLP on the radar data, we were able to classify metal and paper reflectors correctly in 87.66% of the runs. For the second configuration, we changed the reflectors to a rectangular shape made of metal or plastic. Again MLP produced the highest classification accuracy (81.06%). For the last configuration, we tried to classify between different shapes of the reflectors, so we selected tetrahedron and rectangular shape reflectors, both made of metal. MLP still produced the best classification result (82.2%). In summary, we are successful in classifying reflectors made of different material as well as two shapes of the metal reflectors. A more in-depth study of the radar’s capability in classifying different types of material can be found in [6].

## 5. Conclusions and Future Work

In this work we have developed an experimental methodology for a distributed multi-modal system for surveillance and tracking using radar and supplementary sensor data. Our research goal is to develop a prototype sensor network using commercial off-the-shelf components and to provide innovative solutions for detecting, tracking and classifying different targets and events under conditions with high noise and clutter [15]. Our distributed system supports a pervasive sensor network approach and it consists of a suite of radar transceivers, supplemented with acoustic, vibration, and light sensors. Note the results presented in this work show the basic capabilities of different sensor modalities in an integrated wireless network platform. The experiments with our low-cost sensors show the feasibility of detecting, tracking and classifying different types of targets. More specifically, with the integrated sensor network we are able to differentiate between the same model trains mounted with different reflectors (radar), two different model trains (vibration and acoustic), as well as the same model train with different loads (vibration). The major results of our studies are summarized as follows:

1. The introduced wireless radar sensor detects the range and velocity of targets with good precision, as confirmed by cross-validation measurements.
2. Radar transceivers, in conjunction with vibration and acoustic sensors, can be used effectively to classify different types of objects. The radar can classify various types of material properties and shapes, while the acoustic and vibration sensors can differentiate among objects of different weight and movement characteristics. Acoustic and vibration sensors can indicate an approaching target even when it is not within the field of view of the radar or optical sensors.
3. Integration of radar sensors with a range of wireless sensor motes is an innovative step toward building robust decision support based on a pervasive sensor network for surveillance and tracking in various practical scenarios.

It is important future task to explore scenarios when the multi-modal aspects of the sensor system is properly harnessed [16]. In order to improve the portability and autonomy of our system, we are currently testing our radar-motes with on-board power and signal generation chips. In future research, we plan to experiment with more complex radar tasks and advance the described sensor fusion and target identification, as well as we will investigate the problem of tracking multiple objects.

## Figures and Tables

**Table 1.** Overview of the radar parameters.

**Parameter**	**Units**	**Minimum**	**Typical**	**Maximum**
Operating Frequency	GHz	24.10	24.125	24.15
Output Power	mW		8.0	
Operating Current	mA	140	175	200
Operating Voltage	VDC		5	
Incident RF Input Power	dBm			+20
Range	m	1	6	65

**Table 2.** Overview of the sensor modes of the distributed sensor system.

**Platform**	**Sensor Type**	**Description**	**Used**

MACOM Tyco	Radar	K-band 24.125 GHz	Yes

SBT80 EasySen	Acoustic	Microphone Omnidirectional	Yes
	Magnetic	Dual axis <0.1 mGauss	No
	Acceleration	Dual axis 800 mv/g	Yes
	Infrared	Silicon Photodiode	No
	Visual light	Photodiode	Yes
	Temperature	Analog	No

**Table 3.** Experimental parameters.

**Type**	**Reflective Profiles (RCS)**	**Distance *r*_0_ (m)**	**Velocity v (m/s)*ṙ***	**Number of Runs**

Train A One cart	Metal or paper tetrahedron/rectangle	0.8, 1.2, 1.6, 2.0	0.40, 0.56, 0.69, 0.74 Forward and reverse	2,000
Train A Multi-carts	Metal tetrahedron	1.2	0.69	40
Train B	Metal tetrahedron	0.8, 1.2, 1.6, 2.0	0.56	160
Tracking	Metal tetrahedron	N/A	0.742	N/A

**Table 4.** Velocity (V) and Range (R) estimation from Doppler radar data.

Velocity (m/s)	0.8 m	1.2 m	1.6 m	2.0 m
Range (m)				

0.74 ± 0.02 [D = 100] *	V: 0.76 ± 0.04 R: 0.89 ± 0.08	V: 0.74 ± 0.04 R: 1.29 ± 0.14	V: 0.76 ± 0.03 R: 1.62 ± 0.14	V: 0.74 ± 0.07 R: 2.01 ± 0.12
0.69 ± 0.01 [D = 80] *	V: 0.68 ± 0.041 R: 0.86 ± 0.08	V: 0.68 ± 0.04 R: 1.25 ± 0.09	V: 0.67 ± 0.04 R: 1.56 ± 0.14	V: 0.65± 0.03 R: 1.97 ± 0.17
0.56 ± 0.01 [D = 60] *	V: 0.57 ± 0.03 R: 0.83 ± 0.07	V: 0.57 ± 0.03 R: 1.26 ± 0.12	V: 0.56 ± 0.03 R: 1.57 ± 0.18	V: 0.57 ± 0.03 R: 1.97 ± 0.19
0.40 ± 0.01 [D = 40] *	V: 0.47 ± 0.01 R: 0.85 ± 0.08	V: 0.47 ± 0.01 R: 1.24 ± 0.13	V: 0.49 ± 0.03 R: 1.61 ± 0.14	V: 0.45 ± 0.02 R: 1.95 ± 0.17

* D indicates the Dial position of the velocity control unit in the range 100 (max) and 40 (min) used in the present experiments.

**Table 5.** Range and velocity estimation during tracking experiment.

**Velocity (m/s)**	**Range (m)**

0.65	2.32
0.75	1.90
0.75	1.79
0.75	1.67

**Table 6.** Classification results using vibration data.

**Classes**	**Number of Runs**	**Classifier**	**Correctly Classified (%)**	**ROC Area**	**Kappa Statistics**

Train A Train B [0.56 m/s]	240	Random Forest	92.5	0.99	0.85
		MLP	97.5	1.00	0.95
		SVM	93.6	0.93	0.96

Three velocities	1,200	Random Forest	91.6	0.98	0.88
		MLP	87.6	0.96	0.82
		SVM	92.6	0.94	0.89

Multiple Railcars	40	Random Forest	92.5	0.98	0.85
		MLP	97.5	0.98	0.95
		SVM	60.0	0.60	0.20

**Table 7.** Classification results using acoustic data.

**Classes**	**Number of Runs**	**Classifier**	**Correctly Classified (%)**	**ROC Area**	**Kappa Statistics**

Trains A and B	240	Random Forest	94.1	0.99	0.88
		MLP	96.7	0.99	0.93
		SVM	97.1	0.97	0.94

Three Velocities	920	Random Forest	80.0	0.91	0.70
		MLP	80.6	0.92	0.71
		SVM	85.7	0.89	0.79

**Table 8.** Classification results using radar data.

**Profile**	**Number of Runs**	**Classifier**	**Correctly Classified (%)**	**ROC Area**	**Kappa Statistics**

Tetrahedron: Metal—Paper	640	Random Forest	85.2	0.91	0.70
		MLP	87.7	0.93	0.75
		SVM	84.1	0.84	0.68

Rectangular: Metal—Plastic	640	Random Forest	80.1	0.89	0.60
		MLP	81.1	0.87	0.62
		SVM	80.0	0.80	0.60

Metal: Tetrahedron—Rectangular	640	Random Forest	74.2	0.82	0.50
		MLP	82.2	0.86	0.64
		SVM	71.6	0.71	0.43
